# The environment as seen through the life of a journal: *Ambio* 1972–2022

**DOI:** 10.1007/s13280-020-01421-w

**Published:** 2020-11-21

**Authors:** Sverker Sörlin

**Affiliations:** Division of History of Science, Technology and Environment, KTH Royal Institute of Technology, 100 44 Stockholm, Sweden

**Keywords:** Environmental governance, Environmental history, Environmental humanities, Environmental journals, Environmental policy

## Abstract

Emerging after World War II “the environment” as a modern concept turned in the years around 1970 into a phase of institutionalization in science, civic society, and politics. Part of this was the foundation of journals. The majority became “environmental specialist journals”, typically based in established disciplines. Some became “environmental generalist journals”, covering broad knowledge areas and often with an ambition to be policy relevant. A significant and early member of the latter category was *Ambio*, founded 1972. This article presents an overview of the journal’s first 50 years, with a focus on main changes in scientific content, political context, and editorial directions. A key finding is that the journal reflects an increasing pluralization of “the environment” with concepts such as global change, climate change, Earth system science, Anthropocene, resilience, and environmental governance. Another finding is that the journal has also itself influenced developments through publishing work on new concepts and ideas.

## An environmental generalist

A great journal is more than its articles. In the history of *Nature* we learn that a journal is also like many other institutions. It has a birth, a childhood, it goes through crises, revolts and reforms, it matures. Sometimes it acquires wisdom and becomes respected, even admired. *Nature* started in 1869, with several failed predecessors in the UK at the intersection of science, arts, journalism, and news. At first it was a magazine. It took decades until it published serious science. It stayed commercial and it often took stands politically (Baldwin 2015, 2020). It did not fully demand peer review in a systematic fashion until it was almost a century old, or just about the time when *Ambio* published its first issue in 1972.^1^

*Ambio* filled a gap. It was a journal about “the human–environment”, as it said in the title; that additional qualifier, “human”, was still required in 1972, as it was in the name of the UN conference in Stockholm the same year, one of the largest international political gatherings ever held by that time in human history (Engfeldt 2009). “The environment” as a concept was not new. It had entered circulation in its new meaning—nature-based conditions for life, especially human life, typically threatened and destroyed by humans themselves—right after World War II (Warde et al. 2018). Especially in the 1960s it started to appear more frequently in everyday parlance and in policy. Scientific journals carrying “environment”, or varieties of the concept, in their titles had just started to appear, but most of the impressively numerous journals that exist today had not appeared in early 1972 and even fewer in 1969 when first plans of what became *Ambio* started to percolate in the Royal Swedish Academy of Sciences in Stockholm, where the journal found its home and where it has remained with its editorial office ever since.

A (far from comprehensive) register of environmental journals, with or without the word in the title, encompasses 350 journals for 2019, almost all with later starting dates than 1972.^2^ Among the few exceptions is *Environmental Research*, started in 1967, perhaps the earliest designated “environment” journal, whose “principal aim” was to “assess the impact of chemicals and microbiological pollutants on human health”, a core area in the formative years of knowledge about the environment. The renaming of what is now *Environment: Science and Policy for Sustainable Development* is a telling story. The journal had started as *Nuclear Information* (1958 to 1964), continued as *Scientist and Citizen* (1964 to 1968) only to gain its new *Environment* title in 1969. Another early bird was *Environment and Planning* from 1969. It is worth mentioning precisely because it did not have its roots in science, but also because of how it expanded into a string of sub-specializations, named simply A (the original), B, C, D, and E. The latter from 2018 has “Nature and Space” as its subtitle and a slant towards geo-anthropology, humanities readings of the geosciences, and the Anthropocene that suggests the continuous conceptual expansion and differentiation of “environment”.

Journal titles indicate the massive broadening of the knowledge base that has been engaged. Yet, several environmental journals in the early years were de facto sub-disciplinary, signifying that “environment” also became sub-specializations within a long list of disciplines. Early examples include *Environmental History* (started in 1970), *Environmental Ethics* (1979), *Journal of Environmental Engineering* (1973), *Environmental Law Review* (1970), *Environmental Archaeology* (1981). Even some of the later arrivals have operated on a similar logic: *Nature Climate Change* (2011), the most highly cited environmental journal only takes on a section of the environment, albeit a big one.

*Ambio* is and always was different. It has had its favored topics over the years and it too has undergone major change, but it always was without formal constraints on what dimensions of “environment” should be covered. It was, as one editorial proclaimed still in the journal’s third decade, a “generalist” journal (Rosemarin 1990). Being an *environmental generalist* journal gives more opportunities to also take a policy relevant role and impact more widely. However, in contrast to the sub-disciplinary *environmental specialist* journals it also has a much more demanding and cumbersome mission, lacking natural boundaries, a given focus, and even an obvious audience.

In the following, I will sketch the half century-long history of *Ambio*. As a journal that has published more than 4000 articles in tens of thousands of pages, this brief Perspective article cannot offer more than a few lines of the rich and important development that it represents. I have tried to focus on the background—how and why did the journal start, and why did it start in Stockholm?—and on what I have perceived as some of the journal’s main topics and themes over the years. These have been guiding questions for my work: What was the relation between science and policy? What was considered relevant knowledge for a journal on the environment and how has that changed? How has the journal been run, editorially and scientifically? How did it balance its regional presence in Sweden and the north with its global reach and ambition? Above all, I have tried to balance content: what did articles cover?, with context: why did the journal navigate as it did?^3^

## *Ambio*: The environment and the academy

On 11 October 1972, the Royal Academy of Sciences decided “to appoint Paul Crutzen, Ph Lic, as a member of the editorial board for AMBIO”.^4^ This was not a very surprising decision. The administrative committee had prepared the case as was customary, with climate scientist Bert Bolin from Stockholm University as a member. In 1959, Bolin had employed this Dutch engineer, a specialist in bridge construction, to assist in computer programming in the Department of Meteorology.^5^ Crutzen, who became a doctoral student under Bolin, was to receive his doctorate the following year, 1973, when he had already published his first article in *Ambio*, on the threat to the ozone layer (Crutzen 1972). In 1995, he received the Nobel Prize in Chemistry with Mario J. Molina and F. Sherwood Rowland for their studies of the decomposition of ozone. But of this no one knew anything in October 1972, just as nobody knew that in February 2000, at a meeting in the Mexican city of Cuernavaca, Crutzen would coin the concept of Anthropocene, proposed as the new official name of our current geological epoch (Steffen 2013).

To select Crutzen for *Ambio* was even expected, in particular if seen in retrospect. In a 50 year perspective Crutzen personifies some of the broad system thinking spirit that with time grew to become a hallmark of *Ambio* and of the Anthropocene idea, and which has been bold and original enough to also win Nobel Prizes. Another Nobel would be earned by Elinor Ostrom, a recurrent contributor to *Ambio*, who won the Swedish Riksbank Prize in Memory of Alfred Nobel in 2009. It was far more surprising that the Academy had established an international journal on the environment. The Academy of Sciences had not exactly stepped forward when environmental issues were discussed before. What had happened?

**Fig. 1:**
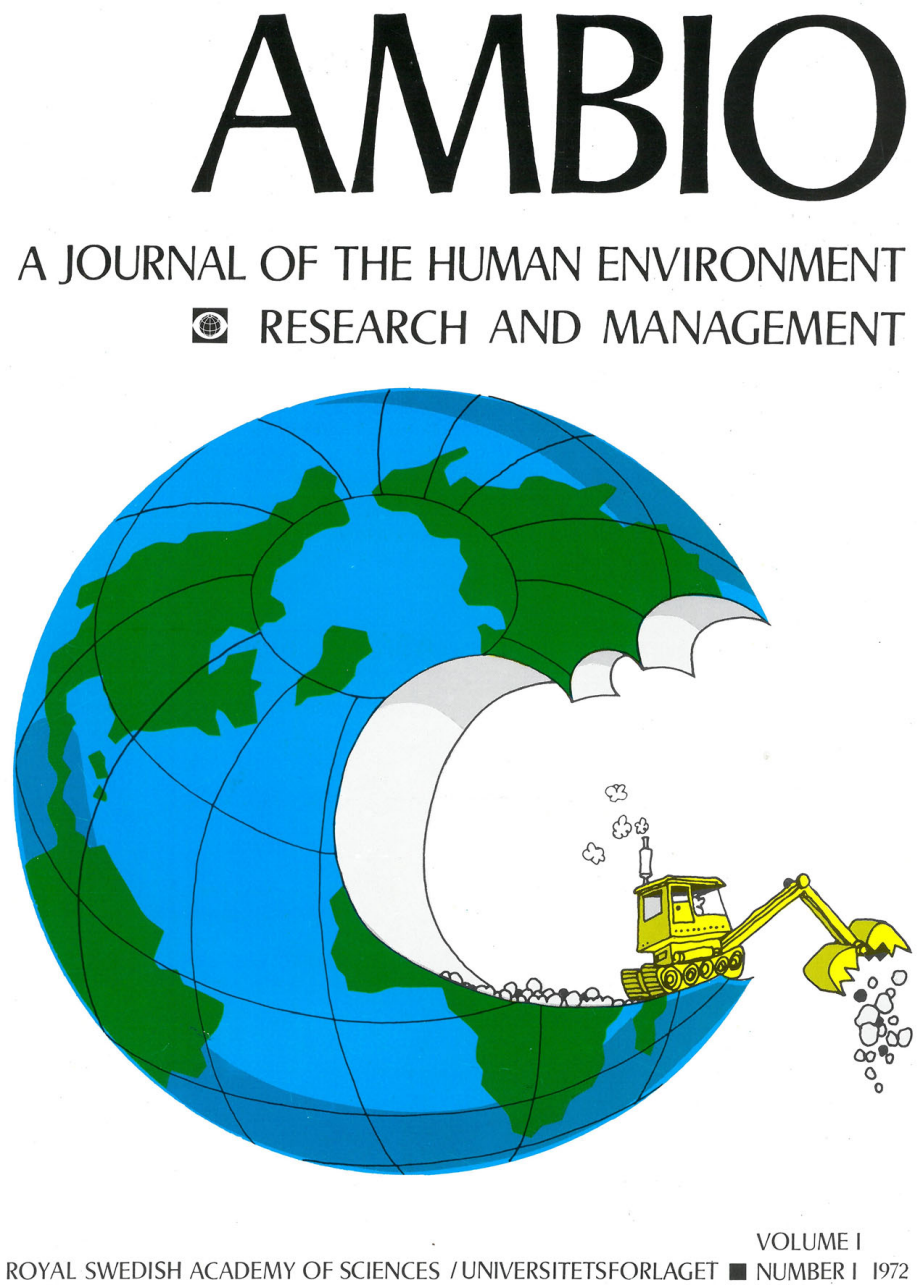
A new journal meets the world—the cover of the very first issue of *Ambio*, February 1972. Cover illustration by Nils Petersson

The name, *Ambio*, was chosen because it signified the environment around us. The word comes from the Latin verb *ambulare*, “walk”, a word that became related with “surroundings”, as in ambient. In Romance languages *ambiente* nowadays means precisely “environment”. The decision to establish the journal was taken in 1969, at the same time as the Academy ended its Swedish language journals for zoology, chemistry and physics, signaling increased international, if not global ambitions. The physicist and journalist Eric Dyring, who was remunerated for a half-time position, began the planning in 1970 and an editorial committee started in 1971. The first issue was published in February 1972. The cover shows Earth being chomped up by an excavator, in essence an illustration of the idea later captured by the word Anthropocene: humanity as the single most important geological force for change (Fig. 1). But perhaps one could also see the proverbial apple of knowledge there, eaten by greed and misused technology rather than consumed to nourish wisdom.

In the decades following World War II, the environment’s key scientific fields lay within what historian Peter J. Bowler, many years later, called “the environmental sciences” (Bowler 1992). He alluded to the geosciences in a broad sense, from geography and geology to geophysics, including oceanography, meteorology and climatology, but also hydrology, ecology and, not least, systems ecology and marine sciences. A great deal, indeed most of it, fell outside experimental physics and chemistry, the traditional fields of the Academy of Sciences, at least as they were interpreted by the Academy’s Nobel Committees. Ironically, for a future Nobel Laureate in the subject, chemistry was not something that Crutzen studied during his years as a computer programmer at Stockholm University. Instead he focused on meteorology and statistics and decided to become a theoretician, also because studies in chemistry required laboratory work that was not compatible with his computing day job.^6^

Perhaps it can be regarded as not just Crutzen but atmospheric research as *an environmental issue* being awarded by the prize. This was actually what his supervisor, Bert Bolin, was in essence doing: tirelessly working to make the atmosphere and climate into a part of a wider understanding of the environment (Bolin 2007), ultimately making him the founding president of the UN Intergovernmental Panel on Climate Change, IPCC and, he too, a Nobel laureate, winner of the 2007 Peace Prize together with among others Al Gore. In turn, Bolin had been inspired by *his* supervisor, meteorologist Carl-Gustaf Rossby, who returned to his Swedish homeland in 1947 after a successful career in the US. In 1956, the same year that Bolin received his doctorate, Rossby had written that humankind was conducting “a unique experiment of impressive planetary dimensions” as it “in just a few centuries, consumes fossil fuels that have been stored for millions of years”. There “can be no doubt”, continued Rossby, “that an increase in the amount of carbon dioxide in the atmosphere leads to an […] increase in the Earth’s average temperature” words also cited in the Rossby cover story of *Time* in December that year (Rossby 1957; Bolin 1959; Bohn and Sörlin 2013).

In the 1950s, anthropogenic climate change was not yet an “issue” at a societal level, barely even at a scientific level, but in the meteorological research environment of Stockholm University it was already since many years on the way to becoming one (Bohn 2011). Rossby wrote his prophetic words about climate change just before his sudden and all too early passing. Nor could the Academy of Sciences fully take them in; when Rossby returned to Stockholm, he was elected as a foreign member of the Academy, but had no leading role and remained somewhat of an outsider. Like Crutzen, Rossby had not studied chemistry or physics in the classical manner recognized by the Academy’s members. As a young student, he had heard Vilhelm Bjerknes, the Norwegian geophysicist, lecture at Stockholm University College and, as soon as he had an opportunity, he went to work at Bjerknes’ meteorological institute in Bergen, then to Leipzig, and eventually to the United States with a letter of recommendation from Svante Arrhenius in his pocket. Theoretical geophysics was calling him, and another type of chemistry, with the entire planet as a laboratory (Sörlin 2015; Fleming 2016).

The Academy of Sciences did not play a prominent role when environmental issues started to appear on the international agenda in the 1950s and 1960s. True, there was a long-standing involvement in issues of nature conservation since conservation legislation had been established in Sweden in 1909, which was early by European standards. The Academy’s nature conservation committee, which advised the Swedish Government, was dedicated to individual cases of nature conservancy, such as the Öland *alvar*, the big rivers of Lapland, or central Sweden’s meadows, often small-scale nature objects, at the level of farms, fields and forests. “Nature” was the word that was used, not the more modern sounding “environment”, with its connotations of pollution, industry and misuse. But an interest in nature was no guarantee for responsiveness to environmental issues, particularly not on a planetary scale.^7^

One illustration is Georg Borgström, another Swedish researcher—originally a botanist at Lund University—who was quick to see the potential of environmental issues and became the warning prophet of population growth and the looming food crisis. Borgström also had a career in the USA, as a professor of economic geography at Michigan State University. He was practically ousted from Sweden after a conflict with the packaging industry, whose research institute he headed (Linnér 2003). Borgström advocated recyclable glass, but this was not popular with the institute’s main sponsor, packaging company PLM. Nor was he welcome at the Academy of Sciences when his name first came up in the 1960s. He was elected as a foreign member later in his life, in 1980, when environmental issues had become established and his own international significance was indisputable.

The Academy’s environmental metamorphosis in the years around 1970 required an innovator like Bert Bolin; he brought planetary thinking and the gist of large international collaborations into the organization. Among the officials inside, he had the support of Lennart Daléus, subsequently known as a politician—later becoming party leader for the environmentally minded Center Party—who worked as the Academy’s head of communication for a period and also wrote bits and pieces for* Ambio*. However, the external forces for change were just as important. One was the fact that the Academy’s funding situation changed. In 1972, the state withdrew the privilege to publish the official Swedish almanac that dated since the eighteenth century, which required a comprehensive rethinking of the institution’s* modus vivendi*. Losing a main source of income, the Academy, traditionally introvert, had to seek new partners and friends. Albeit orthodox and aloof, it could not remain unmoved as environmental opinion had been on the rise during the 1960s, to reach a peak moment around 1970. It was in the sixties that the concept of “the environment” really started circulating broadly and also when the early environmental movement emerged, in Sweden and internationally (McCormick 1989, ch 3; Guha 1999). The word’s breakthrough in Sweden can be unequivocally dated to 1963–1964 (Sellerberg 1994), and most Western countries followed a similar timetable.

The environment soon became something to which mainstream politics also had to relate, and in that situation the Academy of Sciences turned out to be useful. In 1968, Prime Minister Olof Palme commissioned top diplomat Sverker Åström with the task of preparing the UN conference that had been decided in the General Assembly and to try to get it located in Stockholm (Linnér and Selin 2013; Kaiser and Meyer 2017; Paglia in press). Many international organizations competed to take positions as the environment rose to prominence as an international policy issue, among those the North Atlantic Treaty Organization (NATO), the Organisation for Economic Co-operation and Development (OECD) and the United Nations Economic Commission for Europe (Schmelzer 2012; Borowy 2019). Another major player was the International Council of Scientific Unions, ICSU. It saw the writing on the wall and established the Scientific Committee on Problems of the Environment, SCOPE, in 1969. In 1970, ICSU’s general conference decided to make environmental research a top priority and, the following year, the Academy was able to announce, in its internal newsletter *KVA Information*, that Sweden and Swedish researchers had a “prominent role” in this development (Sörlin 2018a). The environment was the new project for the future.

More and more arrows were now pointing towards Stockholm. Bert Bolin was unceasingly active and had a leading role in the birth of the Global Atmospheric Research Programme, GARP. His networks were essential when the Academy co-hosted another preparatory meeting for the UN conference in the summer of 1971, on “Man’s impact on climate”. It was organized in partnership with the Royal Academy of Engineering Sciences and MIT in Boston, and was held at a conference center on the island of Lidingö, near Stockholm. At the Academy, the nature conservation committee became the subject of a review and, in the spirit of the times, was renamed the Environmental Committee in 1973, and the Academy’s working group for SCOPE was integrated in the organization.^8^

The UN conference itself was held in June 1972, with Folkets Hus in Stockholm as its hub. Delegations came from 113 countries, many led by heads of state and government, as well as multitudes of companies, public agencies and NGOs. An unofficial but, as it turned out, lively and important “People’s Forum” was organized in the suburb Skarpnäck for stakeholders in civil society and thousands of environmental activists from all around the world, not least the global South (Najam 2005). A standard was set for major global meetings: state power should meet the power rising from below, which often consisted of highly qualified experts and religious and civic leaders.

The environmental engagement was enough of a force to make Stockholm a site of global attention for issues to do with sustainability and climate (Paglia and Sörlin 2021), and it had a lasting influence on the Academy. A donation allowed it to start the Beijer Institute (the International Institute for Energy and Human Ecology) in 1977, which was renamed the Beijer Institute of Ecological Economics in 1991. When the major international environmental programs and institutions were shaped in the 1980s, the Academy had already gained a reputation as an environmental player. It hosted the International Geosphere Biosphere Programme (IGBP) from 1987. In 1988, the United Nations Intergovernmental Panel on Climate Change (IPCC) was founded, with Bert Bolin in a seminal role and its first chair.

The political and institutional forces played a major role for *Ambio*, for the decision to establish it but also, as we shall see, for its subsequent development. But, importantly, the relationship was reciprocal—the journal helped pave the way for the Academy as it sought a new role as an environmental institution. When Mistra, a strategic environmental foundation, made a major investment in research in natural resources in the 2000s, the Academy of Sciences was again an asset through its Beijer Institute, now for a consortium of collaboration with researchers from Stockholm University, the Stockholm Environment Institute and KTH Royal Institute of Technology. With lively support from global partners, the Stockholm Resilience Centre was founded in 2006, boosting Stockholm’s role as an international center for climate and the environment. When the major international science programs were transferred to a new umbrella organization, Future Earth, one of the five global secretariats was located at the Academy of Sciences in Stockholm.

## The first edition

The environment crossed established borders and provided a common set of issues that were relevant for a whole range of disciplines along the entire academic spectrum. The environment represented a broad set of relationships between humans, their societies and their “surroundings” (Benson 2020) and it therefore opened the door to broad, integrative approaches as well. William Vogt’s environment classic, and diatribe, *Road to Survival* (1948)—one of the very first to apply the word in its new meaning—was characteristically organized as a set of looming dangers for the earth and humanity: population growth, soil erosion, industrial pollution, overfishing, overgrazing, deforestation, and a range of others (Vogt 1948). *Silent Spring* (1962), Rachel Carson’s eye-opening book that conquered the world with translations into some twenty languages,^9^ brought yet another major theme: biocides and toxic substances (Carson 1962). The list has continued to grow to include urban sprawl, occupational health, climate change, often broad, complex themes. A look at the agenda of the Stockholm UN 1972 conference is a sobering reminder that the issues under discussion could not easily be dealt with by single discipline specialists, although these of course provided useful information. In Stockholm, climate came on the agenda as an environmental issue, as anthropogenic climate change was gradually becoming the norm of scientific understanding.

It was in that situation of rise and expansion for the environment that the Academy of Sciences in Stockholm made its move and decided to start a journal devoted to this rich and burgeoning topic. The first editorial did so by talking about the environmental challenge facing the world and how responses were needed, not least in making science talk to politics. That was a main goal at the outset, not just to publish new scientific results but to communicate environmentally relevant news and ideas across professional and societal boundaries. *Ambio* was not supposed to be a journal for disciplinary specialists, rather one where specialists could meet. “The news published in AMBIO will be of high scientific standards, and will be directed not only to experts, but also to scientists in other fields and to other interested readers” (Dyring 1972).

Still, in practice, that did not seem to encompass all knowledge. In line with most understanding of what was relevant knowledge about the environment in the first quarter century of environmental awareness, *Ambio*, was “dedicated to recent work in the interrelated fields of environmental management, technology and the natural sciences” (Dyring 1972). Not to humanities, social, or behavioral sciences, and not to the study of the politics, law and cross boundary communication that were presented as the essential methods of progress on the environmental front. With time, and with the general broadening of environmental expertise (Sörlin 2013), that would also change, although *Ambio*’s natural science core has remained.

Environment is a global issue, the first editorial emphasized, but initially the journal would, although published in English for outreach, have a focus on the Nordic countries, including Iceland. One feature with the initial declaration of the journal’s scope is particularly striking: this was a news magazine. Its mission was to make environmental information available. This was not fully in line with what most academic journals do. It was a special effort, designed to meet a growing demand for overview and enlightening information about what was perceived as a massively growing global problematique.

To meet this demand the journal set out with a substantive news section and with an equally sizeable report section, where scientists gave state of the art presentations of current problems at the environmental front, rather than publishing their own latest individual research, although overlaps certainly occurred. The upcoming UN 1972 conference in Stockholm was explicitly mentioned in the first editorial as timely, and in the following, very first article of the journal the Swedish top diplomat at the UN, Sverker Åström was given the opportunity to outline the thinking behind the conference and its ambitions. Åström was, untypically for a diplomat, quite alarmed and far reaching. “[I]t is late”, he wrote, action is needed. His main target was “the doctrine of sovereignty”, which was a “hindrance to the kind of international cooperation which is now required for rational environmental policies” (Åström 1972, p. 4).

To stake out territory in “the environment” and to identify themes of high politics, for example peace and the fight against nuclear weapons (both occurred in the journal in the early years), that were congenial with the advisory, non-partisan roles of science were all in line with what the Academy of Sciences was used to doing. It was a body that had since long left its once active role in doing science in-house and engaging directly in its pursuit through building research institutions and funding them. It had rather taken on a brokering and promotional role to raise the status and influence of science in modern societies with the Nobel Prize in chemistry and physics as its main annual showcase (Grandin et al. 2018). In this branding of the Academy the new journal fitted well. Environment was the emerging paramount issue where science and knowledge were essential for a happy solution. To strategically move in that direction seemed the right step to take, especially for an academy that had been struggling for some time to find a role in a situation where higher education and research grew massively in sprawling universities, some old, some newly established and when academies were sometimes regarded like a thing of the past. The environment was a place to be if you wanted to stay, or become again, relevant in emerging knowledge societies.

## Post-Stockholm: Finding a niche

*Ambio* started on a bi-monthly basis and the following issues of 1972 were heavily marked by the UN conference. Its general secretary Maurice Strong contributed to the then current June issue with a powerful vision of what Stockholm could mean to the world—and “the new direction man must take” (Strong 1972, p. 77). Otherwise fairly typical early 1970s environmental themes filled much of the slim, forty-page issues: marine chemistry, hazardous materials. The Scandinavian, especially Swedish presence was quite overwhelming, especially in the news briefs section where international names were few among the contributors. Later the same year, Sweden’s ambassador to the UN, Inga Thorsson, was interviewed on how the 109 point program adopted in the final declaration from Stockholm was addressed within the UN system. Generally favorably, but already with some noteworthy points of friction.

**Fig. 2:**
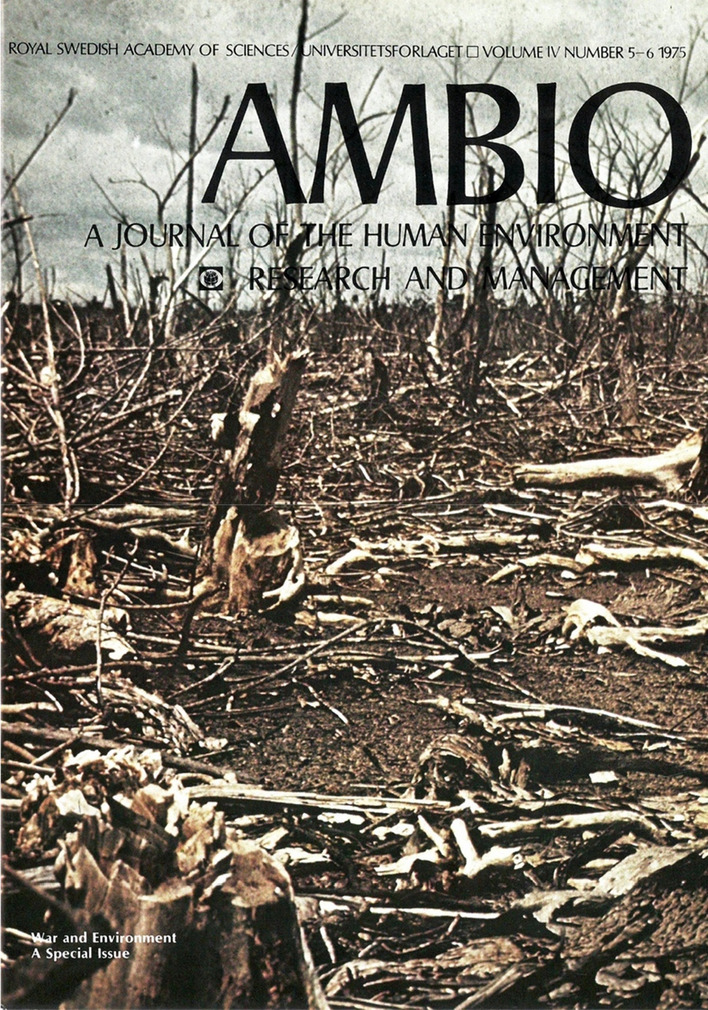
Volume 4, Number 5–6, Special Issue: War and Environment. Original cover caption: Mangrove area sprayed with herbicides several years before the photo was taken. Gia Dinh province, South Vietnam, August 15, 1970. Copyright Arthur Westing

The post-Stockholm process was paid a lot of attention. Inga Thorsson reported again from the UN in 1975, in a special issue on “War and Environment” (Fig. 2). She regarded disarmament negotiations as a de facto work for the environment (Thorsson 1975). Jozef Goldblat, an arms control expert at the Stockholm International Peace Research Institute (SIPRI), discussed “prohibition of environmental warfare” (Goldblat 1975). Environmental problem-solving was almost invariably seen through the lens of legal action and conventions. Signaling this basic trust in institutions and agreements, almost every step on the way taken by UNEP, one of the outcomes of the UN meeting, was reported. But as the years passed, the cheerful spirit of the Stockholm moment tapered off. A decade later, Patricia Scharlin returned to the legacy of Stockholm. She noted that there had been “Three Decades of Concern”, since the formation of IUCN aided by UNESCO in 1948 and the Lake Success conferences the following year, but “Progress Is Still Slow” (Scharlin 1982; cf. Warde et al. 2018: ch. 1).

The relation of UN and other international organizations to the environment remained an active concern of the journal in the 1980s. It waned slowly in the following decades, but returned occasionally and with force at the time of the UN environmental meetings in Rio 1992, Johannesburg 2002, and London 2012, and when the Sustainable Development Goals (SDG) came on the agenda in the years following the financial crisis 2008–2009. The editorial office traveled to the largest meetings and Elisabeth Kessler, editor-in-chief in the 1990s and 2000s, remembers how she and her staff brought copies with them to showcase the journal as a relevant resource for everyone who wanted to stay on top of the environment. Some 11 000 copies of issues 1 and 3 for 1992 were handed out in Rio, Kessler reported to the editorial board the following year, and the same procedure was repeated during the “environmental week” organized by the Swedish Environmental Protection Agency at the World Expo in Seville, also in 1992.^10^ Thousands of copies were distributed for free again at the UN Habitat meeting in Istanbul in 1996 (Kessler, pers. comm.).

This was in line with an active policy of the journal to reach out and become the “go to” journal of the environment in the developing countries, an effort that extended beyond what most science journals would do to promote environmental knowledge and policy in low-income countries. In 1993, Kessler could report more external support, from the World Bank, and from the Chinese Academy of Sciences, supporting a translation of *Ambio* to Chinese that started the same year.^11^ Still, most of the costs had to be picked up by the Academy in Stockholm, and, later in the project, by the Swedish International Development Agency, Sida, that had already supported a wide distribution of copies to developing countries. The Chinese print-run of 2500 copies was mostly distributed for free to libraries, universities, and other institutions across China (Kessler and Zhang 2001). The China project went on for more than a decade, by which time Chinese scientific institutions had developed enough to allow many scientists to find their own way to the journal, as readers and in due course also as contributors.

The social sciences were commonly held in the background, fully in line with the mission statement that the Academy’s “working group for the planning of *Ambio*” had set up prior to its launch. The journal should “concentrate on the natural science and technology sector of environmental protection”, a phrase that was repeated in the journal’s self-presentation for many years. It was also declared that it should reach out widely, to scientists and policy circles “across disciplinary boundaries” but the implicit expectation was that the actual production of knowledge should be conducted along disciplinary lines; at least no desideratum about interdisciplinarity was explicit in the early years. Each issue was, however, multidisciplinary, in the sense that the contributors came from a broad range of fields, and many articles were single-authored, or by small groups.

The mission statement also signals very clearly an explicit foreign policy dimension for the journal that was unofficially national: “A country that wishes to pursue an active and progressive environmental policy is required to spread information and conduct a debate around environmental protection across boundaries”. There is no doubt that the “country” referred to here is Sweden, and in a style quite typical of Sweden’s 1970’s self-perception as a “model” country the document continues to note that there is a large international interest in Swedish research, debate, planning and legislation on the environment, and a journal in English will become a practical instrument to disseminate what Swedish and “other Nordic environmental research” could bring to the wider world.^12^

The de-prioritizing of the human and social sciences was not so much deliberate as it was a default mode of viewing what the environment was all about, and also what most of the Academy of Sciences was all about. The humanities and the social sciences were thin in the Academy, with one class each of a total of ten. But the mission statement did not preclude engagement with the human sciences either. Already in 1972, Jack Hollander, a Berkeley physicist, discussed the role of the scientist and referred explicitly to sociology and philosophy as the legitimate professional fields where issues of responsibility and research ethics were studied. However, Hollander argued, issues of environmental responsibility had to be dealt with by the scientists themselves. To avert damage and disaster, the scientists must abandon their conventional neutralism and non-consequential attitude to the effects of their discoveries and the new technologies that they made possible (Hollander 1972).

Hans Palmstierna, a Swedish medical scholar, introduced to *Ambio* the social differences embedded in environmental issues. He was concerned that the scientific community was so easily engaged in the “outer” environment, while concern for the workers in unhealthy sweatshops and in poison-laden plantations, farms, and fields was almost nowhere to be seen in the scientific literature. There was a lot of research on mercury, asbestos and dieldrin, Palmstierna argued, but little collaboration with those that were affected and sometimes killed by the substances. Is it not strange, he asked, “that we have not taken warning from the diseases and deaths of our fellow human beings who work in the factories and workshops, where so many dangerous substances occur in concentrations that far exceed those found in Nature?” (Palmstierna 1972, p. 110).

With this broad and slightly amorphous remit it should come as no surprise that forces soon appeared that regarded the journal as not scientific enough. The professionalization of the journal became a strong trend, beginning early and emerging alongside its broader policy ambitions. By the mid-1980s, the tensions had grown to open conflict, and some of the science consultants resigned. Science editor Jan Erik Kihlström, a biologist who had been on the editorial board from the early days, declared that the journal must become “more scientific”, while the representative of the Swedish EPA took the opposite view and argued for widest possible range. The tensions were inbuilt, with a board that was largely made up of official representatives of public agencies and funders surrounded by a scientific community that essentially, and increasingly, saw the journal as a publishing window with global reach. During the 1980s, the generalists still held the turf strongly.

In the beginning, instructions for authors were scant and the editorial advisory board was thin. *Ambio* had the appearance more of an environmental affairs magazine, again reminding of *Nature* in its first century. Many years into the journal’s existence there were still no regular principles for peer review. When they start to appear, in 1974, a decision on submissions was promised after between 4 to 6 weeks, with no mention of systematic scholarly peer review. Swedes dominated among the authors, although this started to wane somewhat later in the decade and was considerably less marked by the middle of the 1980s, likely an effect of the shift to non-Swedish editors. This notwithstanding, Sweden would remain the by far most significant contributor of articles over the years, with close to a thousand of the lead authors of 3522 articles (1973 to 2019).^13^

**Fig. 3:**
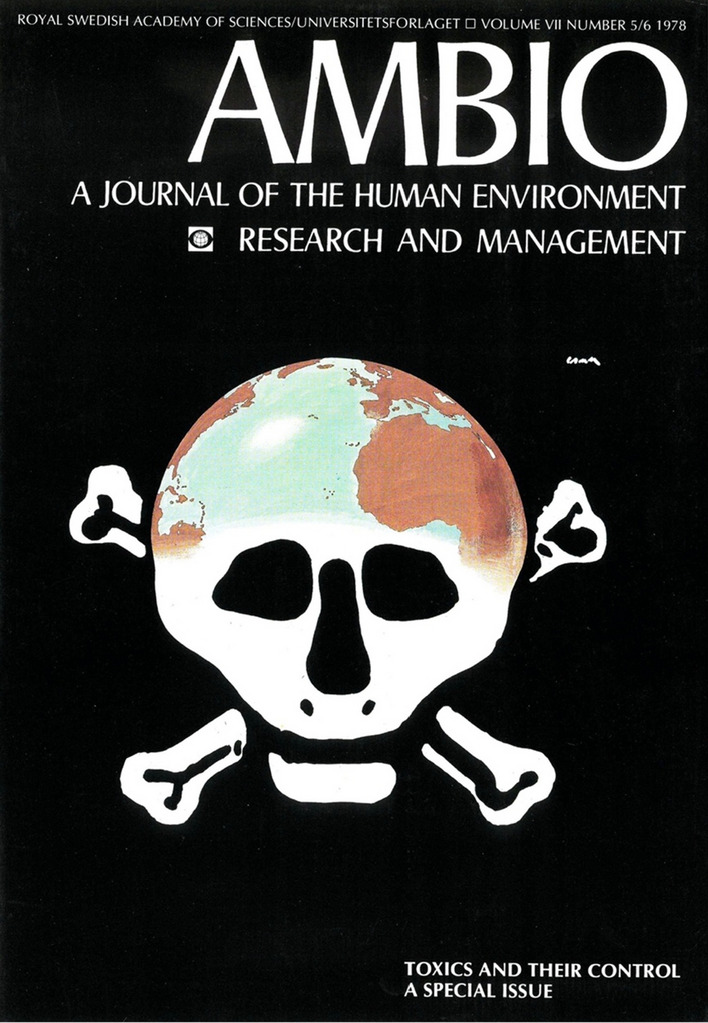
Volume 7, Number 5–6, Special Issue: Toxics and their control. Drawing by Martin Lamm

The first few years of the journal show a lack of clear orientation not unusual for new journals. *Ambio* in the first half of the 1970s is marked by the global environmental moment that it was born in. It apparently wanted to become a journal that reflected environmental policy and its concerns, but at the same time not one that made policy part of its scientific inquiry. Very few articles were based on *research* about policy. The science that was presented was almost invariably the main topics of the early phase of environmentalism: chemicals, toxic substances, and other pollutants—phenoxy acids, lead, dioxins that negatively affected the environment. A recurring contributor in those years was Søren Jensen, a Danish chemist, who had moved to Sweden and researched PCBs and spearheaded the quest to get PCBs banned (Jensen 1972; Spears 2014; Markowitz 2018). A Swedish name that featured was Christoffer Rappe, a chemist at the young and aspiring Umeå University in the north, whose work on dioxins in breastmilk became well known. The subject of pollutants, in both terrestrial and aquatic environments, and policies to deal with them, was one of the strongest and also merited a special issue in 1978, “Toxics and their control” (*Ambio* 1978; Fig. 3).

Already by the late 1970s, the journal had found a more consolidated direction, reflecting the normalization of environmental policy work and also the steady growth of the science across a broad range of disciplines. Over the decades, the Baltic Sea was an empirical foundation for a large number of the articles, obviously reflecting the home base of the journal but also the institutional engagement of several Stockholm-based research institutions in the geographical vicinity of the Academy of Sciences, and also membership in the Academy in many instances. One of the Stockholm-based authors who would become a seminal contributor to the journal was Malin Falkenmark, a tireless, theoretically astute and visionary hydrologist. Regional special issues tended also to focus on marine environments: the Mediterranean, the Pacific and the Baltic Sea. Much later, in 1989, the Arctic became the topic of a special issue, again with a strong focus on ocean and marine topics. On the terrestrial side eutrophication was a major trade, again with water as a strong element.

## The second decade: Diversifying content and scope

In the 1980s, more issues were added to the mix, driven by the epistemic and policy evolution of the environmental agenda. Forestry became a major interest—from issues of soil to deforestation, including the lamented loss of rain forest, with Amazonia as a special case. Food also came in, as did acidification. The international presence was steadily growing, especially the tropics, but also arid zones, wetlands, mountains, shorelines, and mangroves. Still, through regional reports, toxics and pollution were always around, covering in some haphazard and heroic fashion most corners of the planet: “Heavy metals in Greece”, “Mountains in Turkey”, “Mangroves in Thailand”, etc. Chernobyl 1986 and Bhopal 1989 received attention. Thematic issues appeared on the Caribbean (sponsored by UNEP) and India. In relative terms the Scandinavian, and particularly Swedish contributions and topics remained distinctly overrepresented but in absolute terms they now dwindled.

There is a clear sense of “the environment” coming of age. Certainly not in a linearly progressive fashion; things still moved painstakingly slowly. Perhaps “the environment” was not, after all, the only game in town. The Reagan presidency in the United States dampened enthusiasm. At the same time more subjects appeared, shifting the relative proportions of topics as well. Marine mammals were devoted a special issue in 1986. Coral reefs were on the rise and a special issue of the ASEAN region was published in 1988 in cooperation with UNEP’s Ocean and Coastal Programme.

The thematic collections provided in depth coverage and *Ambio* readers apparently liked them; they also helped an essentially Western (if not Nordic…) journal to globalize its presence and to systematically open its pages to new scholarship that otherwise may not have become visible. It was also increasingly an economic necessity. It meant collaboration with existing programs and institutions that stabilized the journal both in terms of input of material and, not least, in editorial capacity with guest editors doing part of the mundane work of soliciting articles and preparing them for publication. Much work was organized in collaboration with subject editors and ad hoc institutional collaborators. They became increasingly popular among the editors as well. List of upcoming special issues was a standing topic on editorial board meetings, along with the financial situation and statistics on sales and circulation. In the 1980s, more than one of four issues was a special issue (thematic ‘special reports’ included). In the 1990s, that number had risen to more than one in three.

A comparatively small share of the articles seems to have emanated from pure basic research, submitted in the regular single article bottom-up fashion. The reasons were also financial. The Academy guaranteed some core funding but in order to fill six issues per year it was in practice necessary to find external partners. Another factor was the constant pressure to find more and new authors. The policy and magazine aspect of the journal, still distinct as the 1990s began, made it less attractive to young and aspiring scientists—contributors were rather more senior and established—and articles published in *Ambio* typically did not count for PhD compilations. To move out of that squeeze, weak finances and lack of scientific prestige, the strategy was to use established networks to expand the pool of contributors and bring in new blood (Kessler, pers. comm.).

There was also a relatively rapid change of editorial leadership in the first two decades of the journal. Eric Dyring left in 1974, later to become science writer for the Dagens Nyheter, Sweden’s largest daily. He was replaced by Ulla Magnusson, an intern from the *Ambio* office. In 1978 Jeannie Peterson, another *Ambio* insider with a US journalism degree and some environmental studies in Stockholm started a five year term. After her came Don Hinrichsen, a US-born environmental journalist and science writer. He too studied environmental science (marine ecology) at Stockholm University when he was hired to the post. Like his predecessors after Dyring he had a weak position in the Academy and did not adopt any decisive new line for the journal but he did establish stronger ties with the World Resources Institute in Washington DC where he went for his next job; the WRI had by then already become a standing presence in *Ambio*. Richard J. Litell, another American, also with WRI links, made a short presence as editor-in-chief during 1985.

Next in line was Arno Rosemarin, a Canadian environmental biologist. He set out in 1986 with an expansive agenda, moving from six to eight issues per year. It brought energy and new readers but it also accelerated the need for external partnerships, and the finances were not strong enough to sustain his ambitions. During the 1970s, *Ambio* had been published by Universitetsforlaget, an Oslo-based university press with a modest profile. This was perhaps too modest, and also too provincial to serve the journal’s global ambitions. In 1979, the Academy dropped the collaboration and instead signed a contract with the then comprehensive Pergamon Press, already used by other journals in the Academy and a major distributor for the United Nations.^14^

Owned by the legendary but erratic and unpredictable Robert Maxwell from his Oxford mansion, Pergamon, despite its strong brand, offered frugal conditions and little support. Editors in the Pergamon group were under strong pressure to deliver financial results and Rosemarin (pers. comm.) recalls bizarre scenes at his annual Oxford visits. As the Pergamon empire started to crumble in the aftermath of the European revolutions in 1989 and Maxwell’s experimentation with *The European*, Rosemarin’s skepticism grew. A couple of years later Maxwell’s economic demise became ever more apparent, followed by his sudden death during a sailing trip in the Caribbean in 1992. By then the journal had already switched to Allen Press in the United States that provided *Ambio* with a more stable publishing platform.

**Fig. 4:**
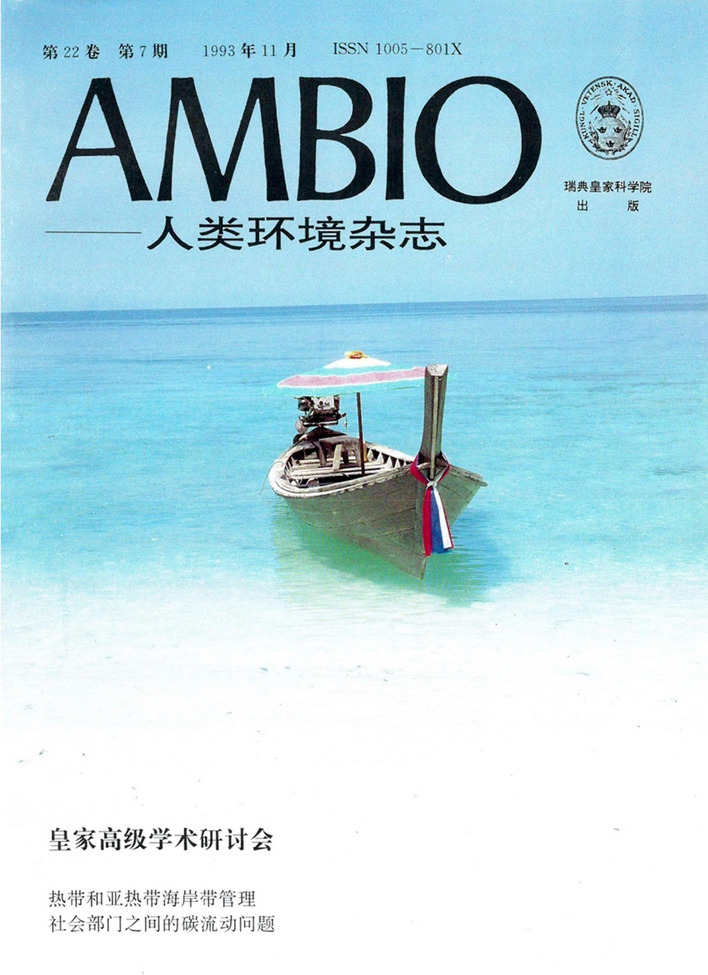
Starting in 1993 and for more than a decade *Ambio* was translated to Chinese. The Chinese print-run of 2500 copies was mostly distributed for free to libraries, universities, and other institutions across China

Most significantly, Pergamon had not been able to increase circulation and global reach, which after all had been *Ambio*’s expectation. Paid subscriptions had started at 2000 (1974), reached 3200 by 1976, a number that did not change significantly during the following 15 years; the single best year was 1983 with 3600 subscriptions. In the 1990s, circulation would grow, but not so much through paid subscriptions but from special issues, Sida-funded distribution support, and the 2500 copies in Chinese (Fig. 4). Submissions grew in the 1980s, but only slowly. In 1997, the editorial committee was informed that the rejection rate had risen to 65% the year before.^15^

Under Rosemarin, who had just left an ongoing scientific career, *Ambio* was more apt to navigate the Academy as an institution than his predecessors. This facilitated his move towards a higher science profile, with more research based content. Many of the policy ambitions, however, were still standing, not least in his carefully crafted editorials, often critiquing the slowness of environmental reform and the need for action. In 1991, he handed over to Elisabeth Kessler, who remained editor-in-chief until 2010. Kessler came from editorial work in the medical sector and brought some of her networks from that sphere. Her long tenure stabilized the journal. Her follower, Bo Söderström, with a biology PhD and a background in science editing and popular science has stayed for one full decade so far and now moves with *Ambio* into its sixth.

## In-house networks and global reach

Both Rosemarin and Kessler benefitted from the significant growth within the Academy of work linked to environmental issues. New institutions, co-located with the Academy, were formed around the Arctic (1984), human ecology (the Beijer Institute, 1977), the global science program IGBP (1987) and the circulation of scientists, workshops, and projects that in one way or the other were relevant for *Ambio* grew steadily.

This distinctly site-specific context of an otherwise fully global journal is one of the strongest features of its development until well after the turn of the century 2000 and still today (Carl Folke pers. comm.). In addition to collaborations with Baltic Sea research groups in Stockholm that had started already in the 1970s, links with neighboring Stockholm University strengthened across more disciplines. As late as in 2006, the formation of the Stockholm Resilience Centre added yet another eager partner. The SRC had strong links to the Beijer Institute, the Stockholm Environment Institute that had been founded by the Swedish government in 1989, and the Academy itself.

This may seem as an inbuilt tension, but since the institutions worked on global issues and were well connected to international networks it was rather a synergetic situation that emerged. A global interest had been there from the early years. The trend had become more visible in the early 1980s with an influx of associate editors with an international background. Jeannie Peterson became editor-in-chief, and as such edited a special issue on “nuclear winter” (1982). Lani Sinclair, Tensie Whelan, Melissa Holloway, recruited under the string of American editors pursued editorial work, solicited articles, planned special issues, and organized the breadth of the international reports for each issue. They also contributed, as staff writers, their own pieces, often in formats that added to the accessibility of the journal and assumed a “global” reader. The relative shrinking of Scandinavian topics came naturally with the changing demography of the journal. A sense of globality took command, as the era of globalization took off.

These changes were seen under Peterson, Hinrichsen and Litell but were continued under Rosemarin and Kessler. It changed the flavor of the journal. Its global mission, always declared, became more borne out in words. It also meant that the semi-official Swedish tonality that *Ambio* had carried from the outset, reinforced by the fact that the editorial board was largely populated by representative of Swedish public agencies for research, environmental protection, and public health well into the 1980s, wore off gradually.

Another feature became ever more significant, namely that the institutions of the Academy formed an inner circle of collaboration. Since the journal was not the baby of any particular one of its ten classes, the independent scientific institutions that dwelled in its premises turned out to be useful partners. These in turn found a household publication outlet with a global reach highly convenient and could find the money needed to pursue the task. They also had the scientists that could provide the content, still a badly needed asset to fill a journal whose impact did not yet bring any large number of spontaneous submissions. At one point under Rosemarin's editorship the journal decided to expand to eight issues per year. Publication later reverted to six, until two extra issues were added again in the 1990s. Predictably, special issues soon started to appear on the polar regions (1989), on ecological economics, on Global Change (the mission of the IGBP office). Other initiatives came through the Academy’s environment committee, an institutional outcome of the major remake of the conservation/environment profile dating back to the early 1970s. Kessler served on it and its experienced chairman was meteorologist Henning Rodhe, drawn from the same department at Stockholm University that had seen Rossby and Bert Bolin move atmospheric and climate issues to the very top of the agenda. She found him a good and reliable partner. Another one was the young and rising Carl Folke who energized the Beijer Institute as it turned more decisively to ecological economics in the 1990s (Kessler pers. comm.; Folke pers. comm.).

**Fig. 5:**
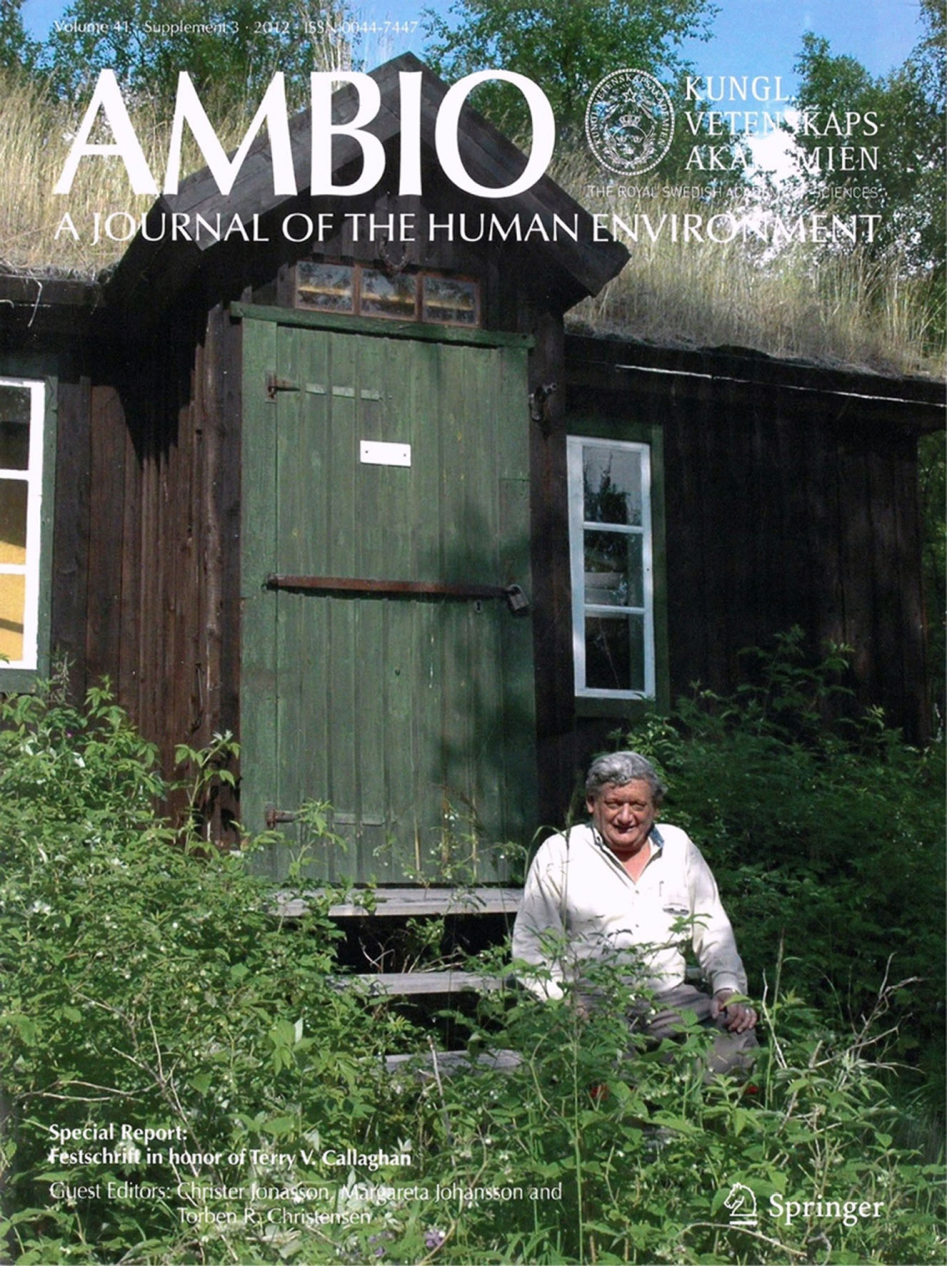
Volume 41, Supplement 3, Special Report: Festschrift in honor of Terry V. Callaghan. Original cover caption: Terry V. Callaghan relaxing in front of the Jieprinkiedde field hut on 19 July 2005 after inspecting damages to the surrounding birch forest following the insect outbreak in 2004. Photo by Torben R. Christensen

In the 2000s, special issues became even more frequent. Several were on the Arctic, one on the “thawing Arctic” in 2006, one on climate change with a Greenland focus. The latter was based on a so-called Royal Colloquium, meetings held almost annually under the chairmanship by his Majesty the King of Sweden, a keen promoter of environmental issues, and often with the editor Elisabeth Kessler taking care of opportunities to get contributions for the journal. These meetings were typically coordinated by Anders Karlqvist of the Polar Research Secretariat. The many polar initiatives over the years contributed to making Terry Callaghan, the tireless leader of the Abisko scientific station in Swedish Lapland—founded in 1903, ceded to the Polar Secretariat in 2011 (Bernhard 1989; Sörlin 2018b)—the most active contributor to *Ambio* over the years, with no less than 43 publications to his name (Fig. 5).

It is hard not to see a pattern to these initiatives that were cumulating over Kessler’s long editorship, namely that bigger initiatives and special issues were rooted within the Academy itself or institutions co-located with it. This may have reflected the relative lack of interest in the journal from the world at large. Submissions were steady but not on the level to sustain eight issues with more pages added over time. It was relatively poorly cited, and remained so until after 2000. *Ambio*’s most highly cited articles of the entire 50-year period are typically from the last 10 to 15 years, with Will Steffen, Paul Crutzen, and John R. McNeill’s “Are we now living in the Anthropocene?” (Steffen et al. 2007) article as the all-time leader, with a strong, but far from sensational 1200 citations in the Scopus index, and more than 3000 on Google scholar.

## Change: Climate and global

It was in the 1980s that climate change started to make itself heard in the journal. Anthropogenic climate change had been an item on the UN 1972 agenda, duly prepared by meetings co-organized by scientists and institutions in Sweden and MIT in the United States (SMIC) (Wilson and Matthews 1971). In UNEP it had a small place, however, and the whole issue did not manage to compete for institutional space in the UN environmental and organizational structures (Borowy 2013; Conca 2015; Macekura 2015). Climate had a very different background story, based in atmospheric physics, which had never been central to environmental issues in their conventional sense, and its scientific infrastructures with monitoring systems and deep Cold War roots did not sit well with environmentalists focusing on public health, pollution and injustices inflicted on Third World populations (Warde et al. 2018, ch. 5).

In the 1970s, the issue could still also be seen as scientifically unresolved, with some of the major research programs and committee work still ongoing, until the National Academy of Sciences committee in the US chaired by Jule Charney—with many networks in Stockholm and the Academy, especially Bert Bolin—unreservedly established in its 1979 report that climate change was real, anthropogenic and required decisive policy action (Charney et al. 1979). The report passed relatively unnoticed, not because it was dismissed, but because the danger was not imminent, and because, perhaps most importantly, environmental expertise and environmentalists moved in other circles and already had such a long list of issues to consider that the vague and distant climate change could not climb on their agenda.

One might have imagined that Bert Bolin, who had masterminded the transformation of the Academy into an international hotspot around 1970, should have used the new journal, and the editorial board position of his protégé Paul Crutzen to launch his climate change research programs, as did so many of his Stockholm scientist peers in the marine and ecological sciences. He did not, perhaps because at the time the environmental context was not where his audience was. They went other places with their significant research and their modeling, a topic that was not much addressed either in the first decade of the journal. The climate change community also had their own Stockholm-based journal, *Tellus*, founded by Carl-Gustaf Rossby in 1949 where an early formation of what we now know as climate change science took place (Bolin 1999).

Climate change had been mentioned in the 1970s, but mostly in passing. It did not receive articles or even editorials. When it occasionally became visible, it was in the form of reports from meetings and projects. A notable exception was in 1975 when anthropogenic climate change was brought up decisively by Stephen Schneider and Roger Dennett, but even then, the topic was conditioned by another more pressing matter: energy. Climate was one of the potential drivers of a shift towards “wind, water and solar” (Schneider and Dennett 1975). The year before climate had been addressed, twice, but again with a conditioning factor, this time population. Was mankind polluting the atmosphere (Landsberg and Machta 1974)? Could the “population explosion” shake climate (Schneider 1974)?

Climate also popped up in brief notes by staff writers, rather than being pushed in articles by climate scientists. Tensie Whelan reported on the later famous Villach conference on climate change in Austria in 1985 (Whelan 1985). The following year, Johan Åshuvud, at the Stockholm School of Economics, reported on a conference addressing possible greenhouse effects in Sweden. There was some concern, more research was needed to gauge the magnitude of the effects (perhaps they would not be all that bad, agriculture would likely be more productive…). Research was also required in order to be prepared for possible future action—not precisely any sense of urgency (Åshuvud 1986). The same year cloud seeding was discussed (Thompson 1986), somewhat surprisingly still a topic despite its already well-documented uselessness (Harper 2017). A report from a research program led by Bolin was reviewed, favorably, in 1987 by Norwegian physicist Hans Økland. He thought it was important that the complex issue was explained for laymen. Such was the approach. To *Ambio* and its readership the whole issue was clearly marginal. The following year was the long hot summer of 1988 when Jim Hansen testified before the US Congress—“Climate change is real”. But that too was an event that did not bother the *Ambio* editorial offices much.

## Earth system beginnings

The game changer was another one and with profound significance for *Ambio*, and in due course for the world. It also started small, as a set of one-page information briefs from a new office called the IGBP. The International Geosphere Biosphere Programme was a major step forward following the Man and the Biosphere Programme that had been started even before the UN 1972 meeting and that inherited its name from the Russian biogeochemist Vladimir Vernadsky’s concept *biosfera*, first published in 1926 and in French a few years later (Vernadsky 1929; Oldfield and Shaw 2006). (MAB had in itself merited the very first *Ambio* special issue in 1975.) The briefs were signed by Thomas Rosswall, a Swedish microbiologist, who had been hired by ICSU to set up the offices of this new program. Their offices were in the Academy of Sciences main building in Stockholm, and literally one set of stairs away from the offices of *Ambio*.

The name used for this new approach was ‘Global Change’, a term that started to float around in the increasingly globalizing decade of the 1980s and merged with the environmental agenda (Malone and Roederer 1985). A study of Global Change had been launched by ICSU during its 21st General Assembly at the University of Berne in Switzerland after the topic was first introduced at the 20th General Assembly of ICSU in Ottawa in 1984. In 1986, Arno Rosemarin introduced it in *Ambio* (Rosemarin 1986). The central idea, articulated by Howard T. Odum in an article in *BioScience* (Odum 1982), was that significant dimensions of the environment that had hitherto been separated—terrestrial ecosystems, atmospheric change, ocean dynamics, biodiversity, environmental history, and, yes, climate change—in reality were closely linked to each other. They needed to be analyzed systematically, meaning as a system of interlinked parts. The fact that they had not was, said Odum, the reason why environmental problems are so disconnected and mismanaged by reductionist ideas. Global Change would speak to a more holistic vision necessary to take on the profound challenges ahead. That was work that required global (it was still not called planetary) presence, and an augmenting of scales, from individual itemized “environments” to an Earth System; the word appears in a brief by Rosswall in 1988 (on p. 357).

The real action was yet to come. The first years were used for planning, in 1990 the research and data collection began in earnest and lasted for the entire decade, and then continued, propelled by the Anthropocene discourse, also a product of the Earth System Science community (Steffen et al. 2015). In the decades following 2000, the Anthropocene conceptualized what may be seen as the epitome of the wave of Earth System science. The Anthropocene discourse, as it has been generated chiefly among the institutionally alert and successful Earth System Sciences (Seitzinger et al. 2015), has rested on an integrative understanding of geophysical, biological and geo-bio-social timescales. This discourse also informs the human–earth relationship and tells us that it has a prehistory with different sites and constellations (Pálsson and Swanson 2016). Increasingly this line of work became visible in *Ambio*.

In the systemic view, climate change became ever more relevant and helped bring about the confluence of these two major strands of environmentally relevant science. Indeed, human agency was also scaling upwards to the integrated systems that the IGBP talked about, and *Ambio* was one of the chosen outlets for this newly formed community. From now on it did not take long for the journal to carry more articles on anthropogenic climate change, and also on precisely those large-scale integrative dynamics that was IGBP’s main concern.

Most of what IGBP worked on was fundamental research, and it remained so when first Chris Rapley (1994 to 1998) and later Will Steffen (1998 to 2004) took over as Executive directors. Yet, in all this there was a clear sense of environmental purpose and engagement. IGBP science was meant to be of use, and the most useful contribution seemed to lay not in the details of management that had been the preoccupation of marine and terrestrial environmental approaches in previous years, but something that would more profoundly rethink the parameters of the human–earth relationship, including to its biological and ecological systems.

## A governance frame of mind

Incidentally, at about the same time, another set of ideas came up that would in an equal fashion contribute to the profound change that ‘the environment’, both as a concept and as a policy area underwent during this period. Not much had been said about this in *Ambio* when Norman Myers, the prolific Oxford-based environmental advisor and popularizer, in one of his many contributions to *Ambio* over the years wrote a comprehensive introduction to the concept of environmental governance. The title already brought the message, “Environmental Challenges: More government or better governance?” (Myers 1988). Myers in his characteristically pedagogic and journalistic fashion dressed up this new concept in four pages underpinned by 37 footnotes, almost entirely with references from the mid- to late-1980s. He sketched the expanding roles of the free market, private enterprise, thousands of NGOs, both old established ones and new ones popping up in the Global South, labor movements, and trade unions. Governments would, indeed should, keep at the back. Civic society, firms, large and small, and flexible parts of the public sector would all move more effectively if left to themselves. They could buy threatened rainforests, alleviate debt by allowing poor countries to pay their loans back with land turned natural reserves.

It was a deregulation agenda for the environment. After all, everyone, both local residents and international capital, would realize, Myers suggested, that the safest way to keep the business thriving and providing jobs would be to keep the ecology working. There were to be no substantive conflicts between economy and ecology. These ideas were explored mostly in other places than the pages of *Ambio*, although they came back there as well. But they grew in significance and they formed the environmental branch of the neoliberal mind frame that was replacing state centralism in most areas of governance and hegemonic social and political thought from the mid-1980s until the financial crisis of 2008–09, after which the tides have changed yet again (Mirowski and Plehwe 2009; Burgin 2012).

Managing or governing the environment became increasingly an issue—how should it be done? The urgency of this question became more tangible in the 1990s with the rise of climate change and Earth System Science as new and major dimensions of the environment, increasingly linked as well and pointing to new levels of truly planetary complexity. These two dimensions also became more and more visible in *Ambio* as well. With scale and scope growing, the close observation on policy as ‘government’, national or UN-led became less pronounced, somewhat in line with the prescription of Myers.

In 1997, the journal offered an entire issue to a centennial of physical chemist Svante Arrhenius, Nobel Laureate 1903, and his famous 1896 “greenhouse” paper, “Ueber den Einfluss des Atmosphärischen Kohlensäuregehalts auf die Temperatur der Erdoberfläche” (Arrhenius 1896). It was orchestrated by meteorologist Henning Rodhe, a long-standing member of the *Ambio* editorial board, Richard Charlson—like Rodhe a member of the climate research community in Stockholm University’s meteorology department whose senior members had been Rossby and Bolin—and Elisabeth Crawford, a Swedish-born sociologist living in France who had just completed a new biography of Arrhenius (Crawford 1996; Rodhe et al. 1997). Most articles were oriented towards past climates or past climate knowledge. Most were also written by scientists, although contributions by the human sciences were included, by Crawford, and by science theorist Aant Elzinga. The issue also carried a piece on the history of climate ideas, especially climate determinism, by Hans von Storch, a German climate statistician, and his countryman, sociologist Nico Stehr. Their cavalcade of pretty unpleasant racist geographical ideas built a strong “Case for the Social Sciences”, von Storch and Stehr argued (1997, pp. 66–71; cf von Storch and Stehr 1999), and the need for social sciences started to be increasingly heard about in things environmental.

**Fig. 6:**
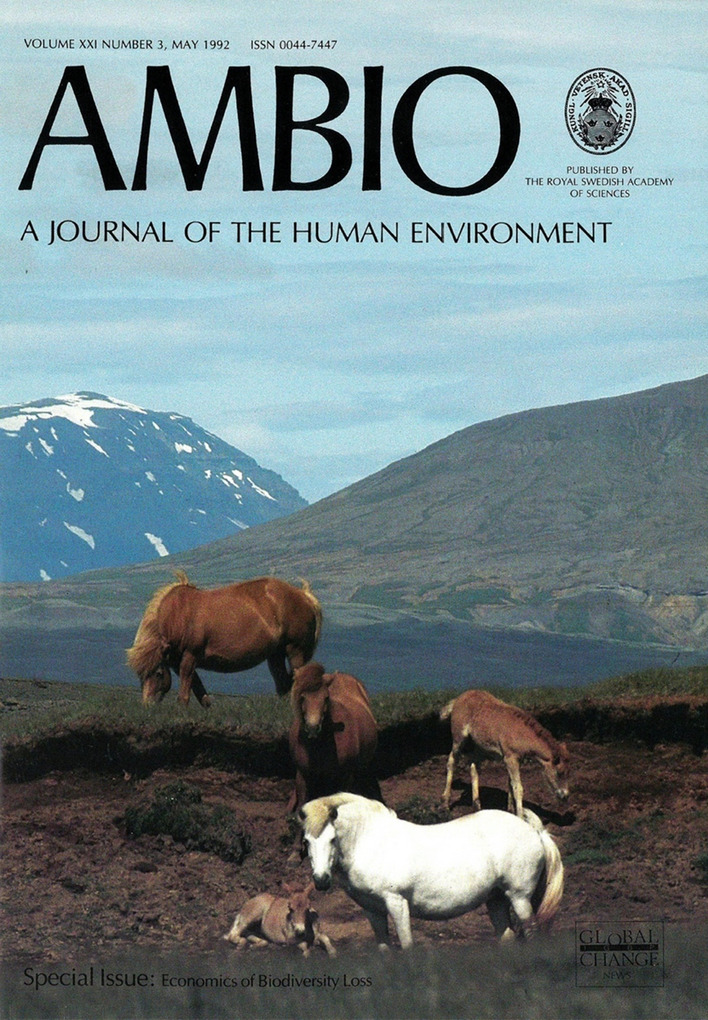
Volume 21, Number 3, Special Issue. Economics of Biodiversity Loss. Original cover caption: Ponies are parts of the human–environment system on Iceland, adapted to but also modifying their life-support environment. The ponies are not only an asset in terms of tourism, but the gene-pool for Icelandic ponies worldwide. Photo: C. Folke

Might economics be part of the governance response? In *Ambio* new relationships between environment and economics were explored more intensely in the 1990s. A major player was Carl Folke, another Swede, who appears in *Ambio* for the first time as author in 1989 in an article with his Stockholm University colleague Nils Kautsky, “The Role of Ecosystems for a Sustainable Development of Aquaculture” (Folke and Kautsky 1989). He was back again in 1992 with a special issue about the “Economics of Biodiversity Loss” (Fig. 6), which he co-hosted with Karl-Göran Mäler and Charles Perrings (Folke et al. 1992). The volume was the result of a research program on the same theme based in the Beijer Institute and brought papers from leading economists and ecologists; Folke himself contributed one on the economics of biodiversity. The following year, Folke and his Beijer Biodiversity program was back with another special issue, with Perrings still on board but with Mäler replaced by Jeffrey A. McNeely (later to become one of the journal’s most active editors and reviewers) and the omnipresent Norman Myers (Folke et al. 1993).

**Fig. 7:**
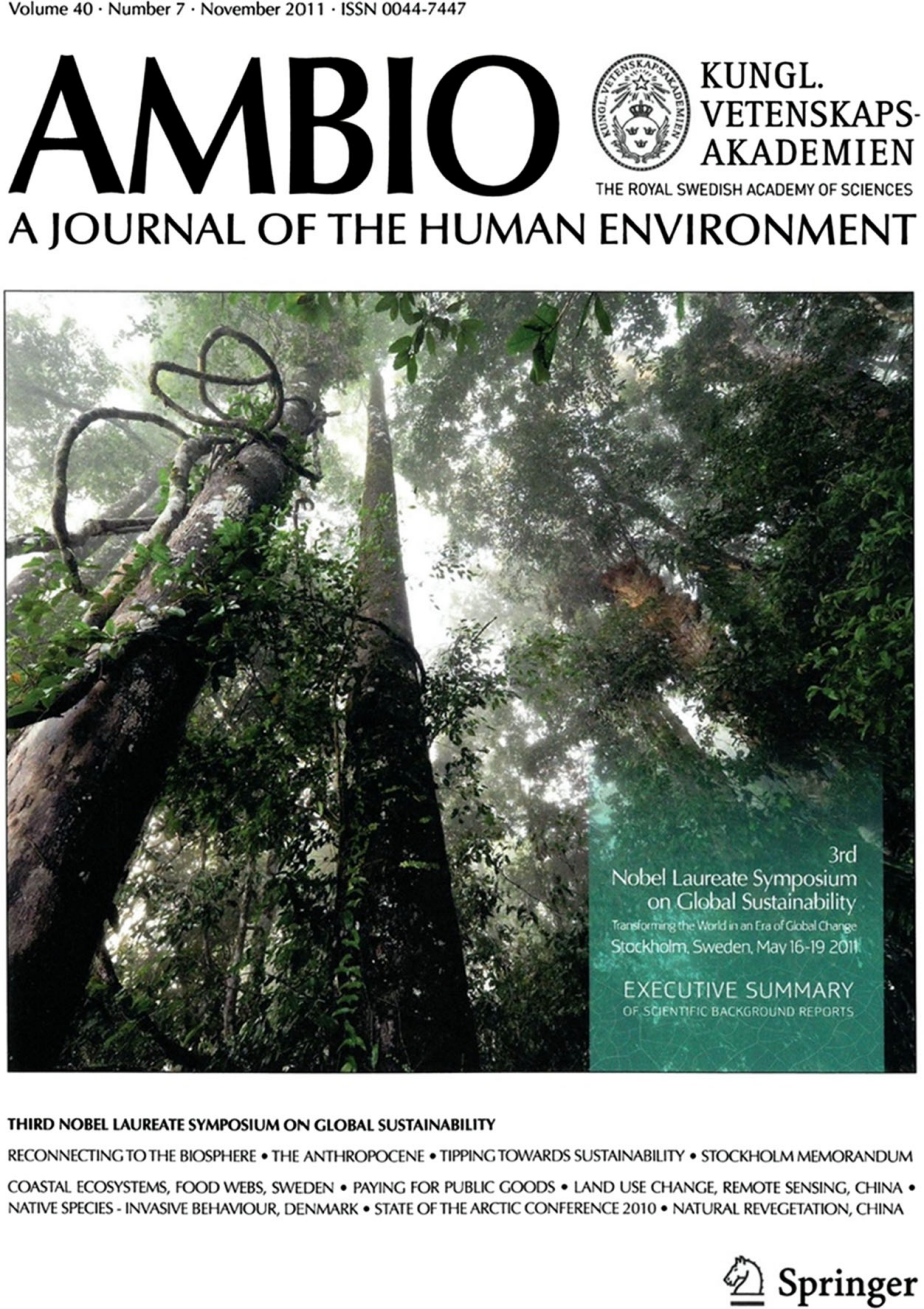
Volume 40, Number 7, Special Section: 3rd Nobel Laureate Symposium on Global Sustainability: Transforming the World in an Era of Global Change. Original cover caption: Dipterocarpus trees and lianas in morning mist in the rainforest of Sabah, Borneo, Malaysia. Photo by Mattias Klum

In retrospect, Folke considers the two special issues essential for the formation of the thinking around social-ecological systems (pers. comm.), and he would remain a frequent contributor to the journal and especially to special issues and reports from large international research programs, on coral reefs, oceans, ecosystem management and several other issues. He co-hosted with Johan Rockström the Nobel Laureate Symposium on global sustainability in 2011 (it became an *Ambio* special issue the same year, Fig. 7; Folke and Rockström 2011), a major organizational feat and perhaps representing a peak moment for the research line that he had nurtured since the early 1990s. Working at the Beijer Institute, Folke’s expanding *Ambio* presence reinforces the pattern of institutional proximity as a factor in the trajectory of the journal. The editorial policies looked favorably at this and as editor-in-chief Elisabeth Kessler has emphasized, not only the physical vicinity but also the conviviality of her in-house partners, Folke at Beijer, Karlqvist at the Polar Secretariat, Rosswall at IGBP, and Rodhe in the Academy’s environment committee inspired and influenced her work. Their support, ideas, networks and other resources made the journal thrive in an institution which otherwise did not engage its leadership much (Kessler, pers. comm.).

## Planetizing the environment

If in 1972 the world, and the Academy, saw the environment rise as a problem that would structure the future, it has in the new century after year 2000 seen a development where environment no longer can hold that center field. This is not because environmental concerns have diminished or that their importance has waned, on the contrary. The environment has become something very different over the half century that has passed. It is entangled with almost everything else. The biggest new arrival is climate change. Another is global change. Both these concepts point to systemic properties of the environment. Earth system science has taken the systemic dimensions even further and expanded the scales of environment, both downwards to nano-levels and upwards to the planetary.

We could speak of a planetization of the environment. It has a longer history but 1972 is a legitimate milestone in what political theorist William Connolly has called “facing the planetary”, referring also to the moral and existential implications of the expanding knowledge horizon of the human–earth relationship (Connolly 2017). Humanities and social sciences have, across the board, elaborated the environmental objects and integrated humans and societies with the environment, since around 2010 under the label environmental humanities (Sörlin 2012; Emmett and Nye 2017). The Anthropocene has become another of those gravitational concepts and one that has drawn interest from both the natural and the human sciences alike (Adeney Thomas et al. 2020; Pálsson 2020).

How has *Ambio* responded to these developments? First of all, it did so by expanding its scope to cover more dimensions and features of the environment. These are too numerous to list, but they encompass anything from environmental law to cultural dimensions of environment, resilience, urban environment. They also bring more detailed levels of inquiry into topics that had always been there allowing for the journal to speak more directly to sub-disciplinary communities of specialists, alongside with the generalists. The journal is by now probably not possible to read in full by a single person, not just because it is already hard for anyone to absorb 160 articles per year (appearing in twelve monthly issues), but chiefly because diversity and depth have both increased to the extent that for most readers the entire content of an issue would at the same time seem both incomprehensible and uninteresting. *Ambio* is perhaps rather an environmental commons where a multitude of communities and individuals could find something for themselves and do it frequently enough to keep staying interested. It has remained a generalist—by giving more to each specialist community.

**Fig. 8:**
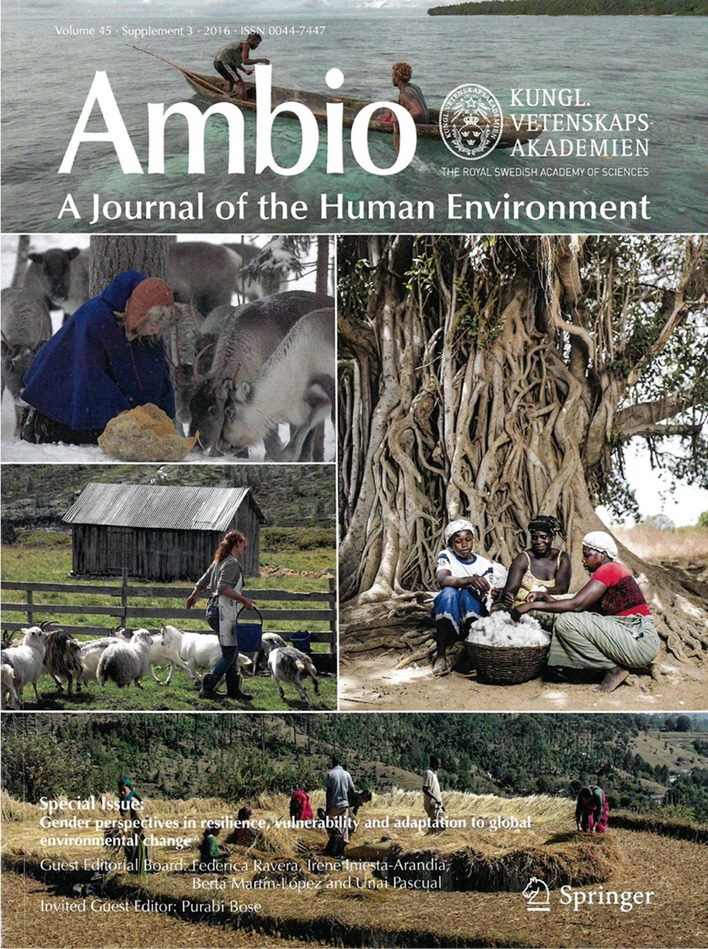
Volume 45, Supplement 3: Gender perspectives in resilience, vulnerability and adaptation to global environmental change. Original cover caption: From the top, left to right: 1. Traditional small-scale fishing over reef in Malaita Province (Solomon Islands, Filip Milovac); 2. Herding woman providing supplement food during reindeer migration in Sami communities (Sweden, Ursula Neussel); 3. Woman deseeding cotton harvest (Burkina Faso, CIFOR); 4. Traditional livestock management run by women in the Valdres Valley (Norway, Berta Martin-Lopez); 5. Communitarian rice harvest in Uttarakhand (India, David Tarrosón)

This combination of intra-scientific professionalization and increased diversity has drawn new readers from ever wider fields, and the interest to contribute has grown rapidly with new topics coming in and impact factors going up. Just as an example, a major issue that turned up forcefully in recent years is gender and environment that was devoted a massive “supplement” volume in 2016 (*Ambio* 2016) (Fig. 8). It had “feminist political ecology” as a keyword to the concluding article and its professed ambition was to explore connections between global environmental change (GEC) with concepts such as resilience, vulnerability, and adaptation. Empirical material came from all over the world, including Sweden and the northern Sami (Buchanan et al. 2016), and feminist studies of ecology with a focus on Mexico and other Spanish-speaking parts of the world. Although somewhat of an outlier, it speaks to changes in *Ambio* that are tangible in many fields. In that regard, the journal reflects changes going on in scholarship and indeed in the world itself. *Ambio* moves with the science and with where “the environment” goes.

The demography of contributors is also changing. They are younger, more widely spread geographically, and represent more fields. In this vein, through practice rather than by principle or program *Ambio* during the Springer Nature co-publication agreement (since 2010) has carved out new directions in the ever growing terrain of environmental science, scholarship and policy. The policy presence is still there and authors are required to provide recommendations in “all articles” which remains an *Ambio* feature, although it is not an easy task to track down their long-term significance. The presence of policy relevant materials may not have shrunk in absolute terms, but considering that the journal in the beginning of the 2020s is at least ten times bigger than it was in 1972 if measured by pages, and probably even more if one counted words, its share is reduced compared to the early years. The environmental policy journalism as a separate feature is all but gone, although some of it remains in Perspective articles.

The growth has instead taken place in the scientific content and in particular in the presence of multidisciplinary approaches. Author groups are larger, representing more specialties, as the complexity of the problems under study is both rising and becoming more acknowledged. The instructions to authors make it clear that the journal “prioritises multi- or interdisciplinary submissions”. However, it also “welcomes intradisciplinary research”. So, *Ambio* has both, but since a long time now it is clearly the multidisciplinary approaches that have had the upper hand. This change in the direction of a more standardized and stringent science journal is manifested in much of the editorial detail as well. All kinds of articles are now peer-reviewed (in the past not all were) and review is double-blind (since 2012). Calls for special issues are open with deadlines twice a year. This has broadened the scope and made closeness to the journal, either institutional or geographical, less of a privilege. The Advisory Board has been considerably widened and now comprises twenty members, covering more fields and for the first time reaching significantly into the humanities and social sciences.

The broadening coverage is also linked to the higher number of articles published which in turn has led to a massive increase of submissions (trebling in the 2010s to more than 600 per year) and to more articles published in monthly issues and with an annual output of 2000 pages or more. Although Allen Press was a relief after the Pergamon regime, the shift to Springer Nature in the last decade has meant a much higher visibility in the scientific community across a wide range of disciplines. The Scandinavian preference that was explicit in the first decades of the journal has increasingly been replaced by a global view although some regional focus areas have been kept (the Baltic and the Arctic in particular).

## Approaching the agenda 2030 decade

It seems obvious, even from a brief review of its first half century, that *Ambio* has reflected the evolutionary broadening of “the environment”. It arrived at a formative moment, when what had been a phase of “conceptualization” of the word in its new meaning during the preceding quarter century was, in the years around 1970, turning into a phase of “institutionalization” of which the founding of environmental journals was a part. It was also a period of massive growth of knowledge and continuous differentiation of what the environment could mean, both in terms of knowledge and policy work. This has continued in the era of “pluralization” that has marked the first two decades of the new century with the growth of concepts such as Earth systems, resilience, and the Anthropocene (Warde et al. 2018, pp. 170–175).

But perhaps a more important, and less obvious, observation is that a broad journal with a significant impact on multiple communities, such as *Ambio*, has also shaped and influenced that evolutionary broadening. Through its knowledge- and policy work, it is part of defining the object and is at the same time busy analyzing, understanding, and, ultimately helping towards better governance and protection. By being precisely an “environmental generalist”, it has managed to do a particular kind of work that few other, more specialized journals could have performed in the same way. Special issues of *Ambio* have over the years moved the boundaries of what the environment “is” and also widened the standards for what counts as relevant environmental expertise. The observation may be most valid for the natural sciences, and less so for the humanities and social sciences, partly explained by the mandate given to the journal at the outset. These more recently recognized realms of environmental and climate expertise have been largely defined elsewhere. But, that said, it should be noted that the human sciences have gained a somewhat higher presence in *Ambio’s* recent years, and given the journal’s trajectory it seems reasonable to assume that this may continue.

As the Agenda 2030 decade has begun and global challenges are bigger than ever—what is the future for a journal with these assets and properties? It is, as the proverbial answer goes, too early to tell… In the ever-growing ecosystem of ‘environmental’ journals there is probably a niche for a handful of critical megafauna as well. A kind of common ground that may not be able to serve all needs—as the magazine of the 1970s still tried to do. But a species big enough to serve its purpose precisely through that common ground that could offer a potentially wide readership, yet at the same time relevant for the epistemic mini-communities that promulgate ceaselessly.

We may perhaps return to *Nature*, where we started, and note that if a journal becomes big and diverse enough it may use its brand to diversify within one’s own kin. As did *Nature* with its sub-species, *Nature Geoscience, Nature Ecology and Evolution, Nature Climate Change*. And as did *Environment and Planning* already since the 1980s. The future may perhaps see an *Ambio Anthropocene*, an *Ambio Humanities*, an *Ambio Arts*, an *Ambio Resilience*, an *Ambio Climate*, an *Ambio Urban*, or some *Ambio X*, *Y*, or *Z* still unknown—new thematic generalists, oxymorons that no one would have thought possible in 1972. Now we can at least imagine them. But what do we know? Environment can go anywhere, and *Ambio*, too.

## Sources

### Royal Swedish Academy of Sciences, archives, Stockholm

Committee for the Conservation of Nature (Naturskyddskommittén), minutes 1965 to 1973; Academy minute books 1967 to 1973; *Ambio* archives: minutes of editorial meetings 1971 to 1997, editorial correspondence 1972 to 2009, financial correspondence, other miscellaneous documents; *KVA information* (newsletter).

### With the author

*Ambio* bibliometric data (articles, topics, keywords, authors, nations, affiliations, citations, collaborations, etc.) based on 3522 *Ambio* publications (3160 articles and 362 reviews) from 1973 to 2019 using data from Scopus, with additional analysis in Excel and Vosviewer, available since 26 April 2020, by Professor Catherine Pickering, School of Environment and Science, Environment Futures Research Institute, Griffith University, Gold Coast, Queensland 422.

### Interviews

Carl Folke 24 September 2020; Elisabeth Kessler 11 August 2020; Arno Rosemarin 26 August 2020; Bo Söderström 30 September 2020.

## Footnotes

1 https://www.nature.com/nature/about/history-of-nature. Retrieved 26 September 2020.

2 https://www.scimagojr.com/.

3 My sources have mainly been the journal itself supplemented with selected interviews of key persons in or near the editorial office in Stockholm, local archival sources, and research literature on the rise of environmental governance.

4 Academy minutes 11 October 1972. Archives of The Royal Swedish Academy of Sciences, Stockholm.

5 http://www.mpch-mainz.mpg.de/~air/crutzen/vita.html. Retrieved 15 October 2020. https://www.nobelprize.org/prizes/chemistry/1995/crutzen/facts/. Retrieved 15 October 2020.

6 https://www.esa.int/About_Us/ESA_history/Paul_J._Crutzen_The_engineer_and_the_ozone_hole. Retrieved 13 October 2020.

7 *KVA Information* 1970–1973. Academy minutes 1967–1973. Archives of The Royal Swedish Academy of Sciences, Stockholm.

8 Information on the Academy of Sciences builds on Academy minutes and on minutes of the Nature Conservation Committee; see reference section.

9 http://www.environmentandsociety.org/exhibitions/rachel-carsons-silent-spring/silent-spring-international-best-seller. Retrieved 2 August 2020.

10 *Ambio* editorial committee, minutes 26 January 1993, paragraph 438.

11 Editorial committee, minutes, 19 January 1994.

12 “Aims of the journal” [in Swedish], document kept in the *Ambio* archives, in box marked “Editorial committee meetings, 1971–1980, 1986–1997”, folder for meeting 26 oktober 1971, agenda point §3.

13 All bibliometric data referred to in the article were collated and analyzed by Catherine Pickering; see reference section.

14 Agreement with Pergamon was signed on 15 May 1979 for a start the following year. A comprehensive documentation of the transition to Pergamon and the relations with Pergamon over the 1980s is available in the *Ambio* archives, box F2:1.

15 Editorial committee, minutes 14 February 1997.
